# Lipopolysaccharide regulation of antiinflammatory tristetraprolin family and proinflammatory gene expression in mouse macrophages

**DOI:** 10.1186/s13104-024-06743-6

**Published:** 2024-03-19

**Authors:** Heping Cao

**Affiliations:** United States Department of Agriculture, Agricultural Research Service, Southern Regional Research Center, 1100 Allen Toussaint Blvd, New Orleans, LA 70124 USA

**Keywords:** Cytokine, Gene expression, Lipopolysaccharides, Mouse macrophage, Tristetraprolin

## Abstract

**Objective:**

Tristetraprolin (TTP/ZFP36) family proteins exhibit antiinflammatory effects by destabilizing proinflammatory mRNAs. Previous studies showed that bacterial endotoxin lipopolysaccharides (LPS) stimulated TTP and tumor necrosis factor (TNF) gene expression, but less was known about LPS effects on TTP homologues and other proinflammatory gene expression in macrophages. The objective was to investigate LPS regulation of TTP family gene and TTP-targeted gene expression in mouse RAW264.7 macrophages using much higher concentrations of LPS and much longer treatment time than previous studies.

**Results:**

MTT assay showed that LPS was not toxic to the cells under LPS treatment up to 1000 ng/mL for 2–24 h. LPS mildly affected the soluble protein content in the cells. qPCR assay showed that LPS stimulated TTP mRNA rapidly but not sustainably with 40, 10, and 3 fold of the DMSO control after 2, 8 and 24 h treatment, respectively. Immunoblotting confirmed qPCR results on LPS stimulation of TTP gene expression in the mouse macrophages. LPS exhibited minimal effects on ZFP36L1, ZFP36L2 and ZFP36L3 mRNA levels. LPS increased mRNA levels of TNF, COX2, GM-CSF, INFγ and IL12b up to 311, 418, 11, 9 and 4 fold, respectively. This study demonstrated that LPS did not affect macrophage viability, dramatically increased antiinflammatory TTP gene expression as well as proinflammatory TNF and COX2 gene expression but had only mild effects on TTP homologues and other proinflammatory cytokine gene expression in the mouse macrophages.

## Introduction

Zinc finger protein 36/tristetraprolin (ZFP36/TTP) is a family of four proteins in mammalian species (ZFP36/TTP, ZFP36L1/TIS11B, ZFP36L2/TIS11D, ZFP36L3) except human lacking ZFP36L3 and birds lacking TTP [1–3]. The function of TTP family proteins is to target gene expression by destabilizing proinflammatory mRNA molecules in mammalian cells. The antiinflammatory TTP family proteins [4, 5] bind to AU-rich elements (AREs) of proinflammatory mRNAs and destabilize the transcripts [6, 7]. Lack of TTP in the knockout mice results in accumulation of proinflammatory cytokine mRNAs coding for tumor necrosis factor alpha (TNFα) and granulocyte-macrophage colony stimulating factor (GM-CSF) and others [8–10], which causes a severe systemic inflammatory syndrome in the TTP knockout mice [11, 12]. On the other hand, upregulation of TTP decreases inflammatory responses in macrophages [13]. Evidence suggests that TTP family proteins are antiinflammatory factors associated with human diseases [12, 14].

TTP gene expression can be increased in mammalian cells by various agents, including growth factors [15, 16], cytokines (TNFα, GM-CSF and interferon-gamma (INFγ)) [9, 13, 15, 17], and zinc [18]. They are also upregulated by plant nutritional extracts from cinnamon and green tea [19, 20] and cottonseed-derived ethanol extracts and gossypol [21, 22].

Lipopolysaccharide (LPS) is a heat-stable endotoxin derived from gram-negative bacterial cell wall. LPS protects gram-negative bacteria against harmful bile salts and lipophilic antibiotics. LPS induces a strong immune response in normal mammalian cells and affects gene expression in cancer cells [23]. LPS from intestinal and oral bacteria contribute to the inflammatory burden and disease activity on patients with rheumatoid arthritis (RA) [24]. However, it is a challenging task to estimate LPS level in human serum. Available data suggest that LPS level in the systemic circulation of humans with healthy conditions or clinical disorders varies widely [25, 26]. Experimentally, LPS has been used as high as 10,000 ng/ml for 24 h treatment [27].

LPS regulation of TTP family gene expression in mouse RAW264.7 macrophages was investigated previously using only one concentration of LPS with short time treatment within 4 h. One study showed that TTP gene expression was rapidly induced by LPS [28, 29]. However, LPS only has minor effects on the expression of the three TTP homologues (ZFP36L1, ZFP36L2 and ZFP36L3) in the mouse macrophages [29]. In another study, TTP mRNA was shown to be peaked at 25 fold of control after 45 min treatment and dramatically reduced to basal level after 60–120 min, whereas TTP homologous ZFP36L1 and ZFP36L2 mRNAs were down-regulated dramatically by LPS (100 ng/mL) [30]. Therefore, the discrepancies in those two studies might be addressed with LPS treatments using various concentrations for longer time treatment.

The objective of this study was to study the effect of LPS on cell viability and gene expression in mouse RAW macrophages using much higher concentrations of LPS and much longer treatment time than previous studies. The targets of gene expression analyses included TTP family genes and TTP-targeted proinflammatory mRNAs such as TNF [9], GM-CSF [10], INFγ [31], cyclooxygenase 2/prostaglandin-endoperoxide synthase 2 (COX2/PGES2) [32], and interleukin 12 (IL12) [33].

## Methods

Mouse RAW264.7 macrophages were maintained as described [22]. The cells were treated with various concentrations of LPS for 2–24 h using 1% DMSO as the control. RNAs were isolated from macrophages and used for cDNA synthesis essentially as described [29]. SYBR Green qPCR reaction mixtures and the thermal cycle conditions were described previously [34]. qPCR primers were designed with Primer Express software and synthesized by Biosearch Technologies (Table 1). The ΔΔ*C*_*T*_ method was used to determine the fold change in gene expression using mouse RPL32 as the internal reference control and DMSO treatment as the sample control [35]. Cell extracts were prepared accordingly [28]. Protein concentrations were determined with the Bradford method (Bio-Rad) [28]. Proteins were separated by SDS-PAGE and transferred onto nitrocellulose membranes in transfer buffer containing 0.1% SDS. The membrane was blocked with 5% nonfat dry milk and probed with the anti-MBP-TTP antibody or anti-ZFP36L1 antibody [28, 36] and detected with the affinity-purified goat anti-rabbit IgG (H + L) horseradish peroxidase conjugate (Bio-Rad). The immune complexes on the membrane were detected with Supersignal Western Blotting Detection Reagent (GE Life sciences) and recorded on an X-ray film. The data represent the mean and standard deviation of 3–6 independent samples. ANOVA were used to analyze the qPCR data with multiple comparisons among the treatments being performed with Student-Newman-Keuls Method with SigmaStat 3.1 software (Systat Software).

**Table 1:** qPCR primer sequence information

mRNA	Accession no.	Amplicon (bp)	Forward primer (5´ to 3´)	Reverse primer (5´ to 3´)
Rpl32	NM_172086	66	AACCGAAAAGCCATTGTAGAAA	CCTGGCGTTGGGATTGG
Ttp/Zfp36/Tis11	NM_011756	70	GGTACCCCAGGCTGGCTTT	ACCTGTAACCCCAGAACTTGGA
Zfp36l1/Tis11b	NM_007564	60	TGCGAACGCCCACGAT	CTTCGCTCAAGTCAAAAATGG
Zfp36l2/Tis11d	NM_001001806	77	GAGGGCACCTCCCAACCT	TGACAGAAGTGTGGTCGACATTT
Zfp36l3	NM_001009549	70	CGAACTGCGTACCCTGTCAAG	GCCAACGCTGTGGAAGGT
Gm-csf	NM_009969	71	CACCCGCTCACCCATCAC	GGAGGTTCAGGGCTTCTTTGA
Cox2/Ptgs2	NM_011198	106	CCACCTCTGCGATGCTCTTC	CATTCCCCACGGTTTTGACATG
Ifnγ	NM_008337	81	TGGCATAGATGTGGAAGAAAAGAG	TGCAGGATTTTCATGTCACCAT
Il12b	NM_008352	79	GACCAGAGACATGGAGTCATAGG	TGTACTGGCCAGCATCTAGAAACT
Tnf	NM_013693	74	GCTGTCGCTACATCACTGAACCT	TGACCCGTAGGGCAATTACA

## Results

### Effect of LPS on cell toxicity

Macrophage viability was measured with MTT assay after cells were treated with the bacterial endotoxin LPS. RAW macrophage viability was increased by 10–35% after cells were treated with a range of concentrations of LPS up to 1000 ng/mL for 24 h (Table 2). MTT assay is a colorimetric assay for assessing cell metabolic activity based on NAD(P)H-dependent cellular oxidoreductase reducing the tetrazolium dye MTT to its insoluble formazan. These results suggest that LPS was not toxic and slightly increased cellular activity under 24 h treatment.

**Table 2:** LPS effect on the viability of mouse macrophages

LPS concentration (ng/mL)	A570nm ± SD (2 h) (%)	A570nm ± SD (24 h) (%)
0	100.0 ± 4.4	100.0 ± 6.6
5	105.6 ± 1.3	132.3 ± 7.5 **
10	98.1 ± 5.7	135.8 ± 8.8 **
20	98.1 ± 5.1	135.2 ± 4.3 **
50	87.3 ± 13.0	129.1 ± 11.0 **
100	80.6 ± 0.2 *	118.7 ± 1.2 *
500	89.1 ± 6.1	113.8 ± 5.1
1000	84.3 ± 5.3	109.7 ± 6.7

Mouse macrophages were treated with various concentrations of LPS for 2 and 24 h. Cellular toxicity was determined with MTT based-In Vitro Toxicology Assay. The data represent the mean ± standard (*n* = 3). “*” and “**” displayed in the Table represent significant difference between the control and the treatment at *p* < 0.05 and *p* < 0.01, respectively

### Effect of LPS on protein content

Protein content, especially soluble protein content could be used as a positive biomarker of cellular activity. The soluble protein content was slightly reduced by 20% without statistical significance in cells treated with LPS for 24 h (Table 3). LPS treatment on total protein content was not significant (Table 3). Both MTT assay and protein determination indicated that LPS was not toxic to mouse RAW264.7 macrophages under 24 h treatment with up to 1000 ng/mL.

**Table 3:** LPS effect on protein content in mouse macrophages

Treatment	Time (h)	Supernatant protein				Pellet protein			Total protein	
		Concentration (µg/µL)	Amount (mg)	Ratio (%)		Concentration (µg/µL)	Amount (mg)	Ratio (%)	Amount (mg)	Ratio (%)
DMSO	24	9.28 ± 1.04	4.64	100.00		16.85 ± 0.21	0.84	100	5.48	100
LPS	2	8.46 ± 0.23	4.23	91.16		25.43 ± 0.18 **	1.27	151	5.50	100
LPS	4	10.52 ± 0.71	5.26	124.35		20.86 ± 0.17 **	1.04	82	6.30	115
LPS	8	10.05 ± 0.90	5.77	109.70		21.59 ± 0.18 *	1.08	104	6.10	97
LPS	24	9.32 ± 0.24	4.66	80.76		19.07 ± 0.39 *	0.95	88	5.61	92

Mouse macrophages were treated with LPS (100 ng/mL) for 2, 4, 8 and 24 h. Protein content was determined with the Bradford method. The data represent the mean ± standard deviation (*n* = 3). “*” and “**” displayed in the Table represent significant difference between the control and the treatment at *p* < 0.05 and *p* < 0.01, respectively

### Effect of LPS on TTP gene expression

**Fig. 1:**
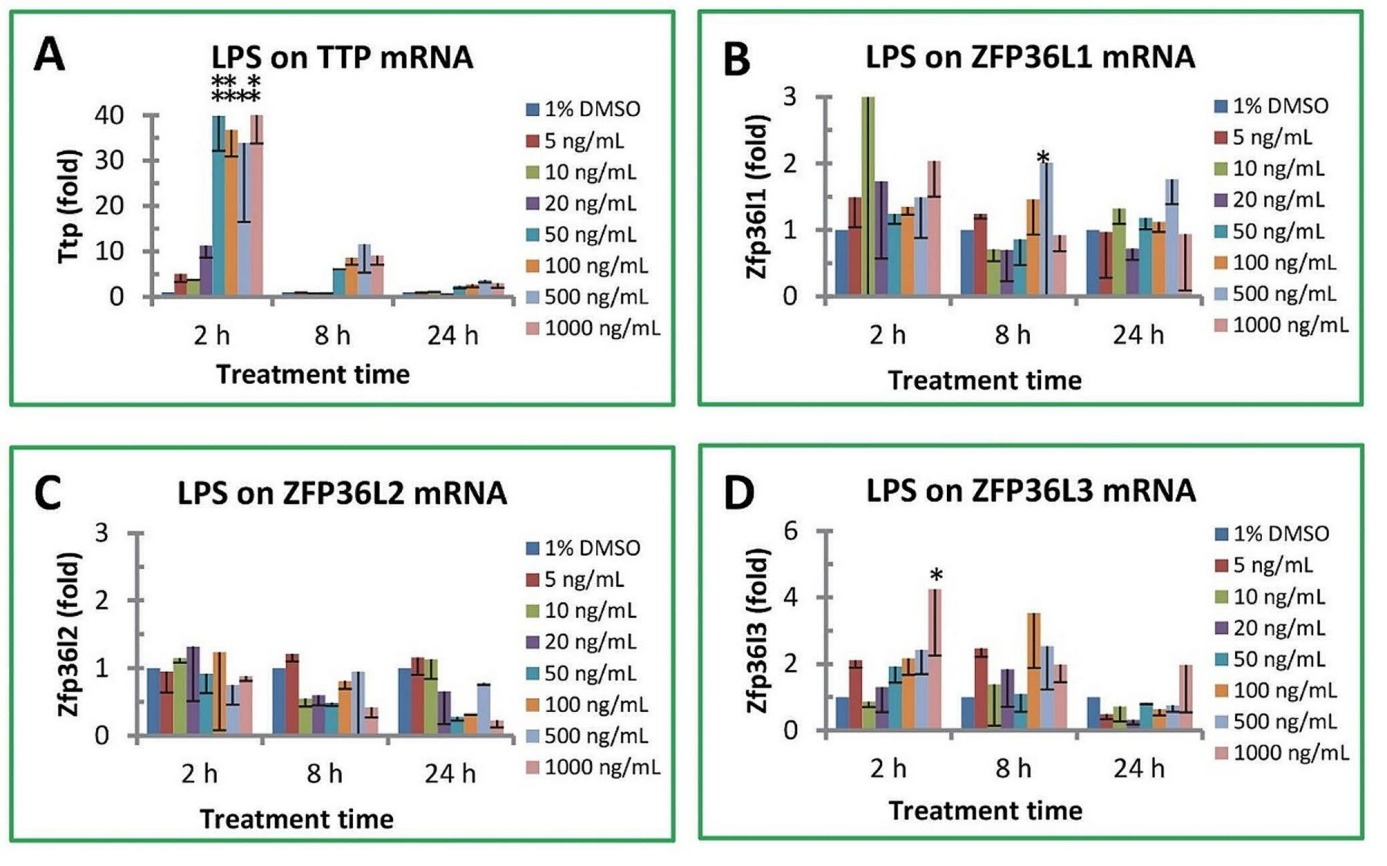
Effect of LPS on TTP family gene expression. (**A**) TTP/ZFP36 mRNA, (**B**) ZFP36L1 mRNA, (**C**) ZFP36L2 mRNA, (**D**) ZFP36L3 mRNA. RAW264.7 macrophages were treated with LPS for 2–24 h. The SYBR Green qPCR reaction mixtures contained 5 ng of RNA-equivalent cDNAs from each sample and 200 nM of each primer. The 2^−ΔΔ*CT*^ method of relative quantification was used to determine the fold change in expression using RPL32 mRNA as a reference mRNA. The data represent the mean and standard deviation of three independent samples. “*”and “**” displayed in the figure represent significant difference between the control and the treatment at p < 0.05 and p < 0.01, respectively. The “C_T_” values of each gene in untreated cells were 17.82 ± 0.81 (Rpl32 control), 24.76 ± 1.12 (Ttp/Zfp36), 26.07 ± 0.35 (Zfp36l1), 24.61 ± 1.03 (Zfp36l2) and 29.20 ± 1.13 (Zfp36l3)

The effects of LPS on TTP family gene expression were analyzed in a time- and dosage-dependent manner (Fig. 1). LPS rapidly and dramatically induced TTP gene expression in mouse macrophages (Fig. 1A). Time-course analysis showed that TTP mRNA levels were increased up to 40 fold by LPS treatment for 2 h; whereas the stimulatory effects were declined after 8 h treatment (up to 12 fold) and 24 h treatment (up to 4 fold). Dosage-dependent analysis showed that LPS induced TTP gene expression in RAW macrophages in an unsustainable way. After 2 h stimulation, TTP mRNA levels were significantly increased approximately 40, 37, 34 and 41 fold by 50, 100, 500 and 1000 ng/mL of LPS (Fig. 1A). After 8 h stimulation, TTP mRNA levels were increased to approximately 6, 9, 12 and 9 fold by 50, 100, 500 and 1000 ng/mL of LPS (Fig. 1A). After 24 h stimulation, TTP mRNA levels were increased approximately 2, 3, 4 and 3 fold by 50, 100, 500 and 1000 ng/mL of LPS (Fig. 1A). However, the increase of TTP mRNA levels in the mouse macrophages treated with LPS for 8 and 24 h were not statistically significant due to large variations of the qPCR data (Fig. 1A).

### Effect of LPS on TTP homologue gene expression

The effect of LPS on ZFP36L1 expression was significantly less potent than that on TTP gene expression in the same mouse macrophages. ZFP36L1 mRNA levels were modestly affected by LPS stimulation and only significantly increased to approximately 6 fold in macrophages treated with 500 ng/mL of LPS for 8 h (Fig. 1B). ZFP36L2 mRNA levels were decreased but not statistically significant by LPS treatment under longer time (24 h) and higher concentrations (50-1000 ng/mL LPS) (Fig. 1C). ZFP36L3 mRNA levels were only significantly increased by high concentration of LPS stimulation under 2 h treatment (4 fold increase by 1000 ng/mL of LPS) (Fig. 1D).

### Effect of LPS on TTP and ZFP36L1 protein levels

**Fig. 2:**
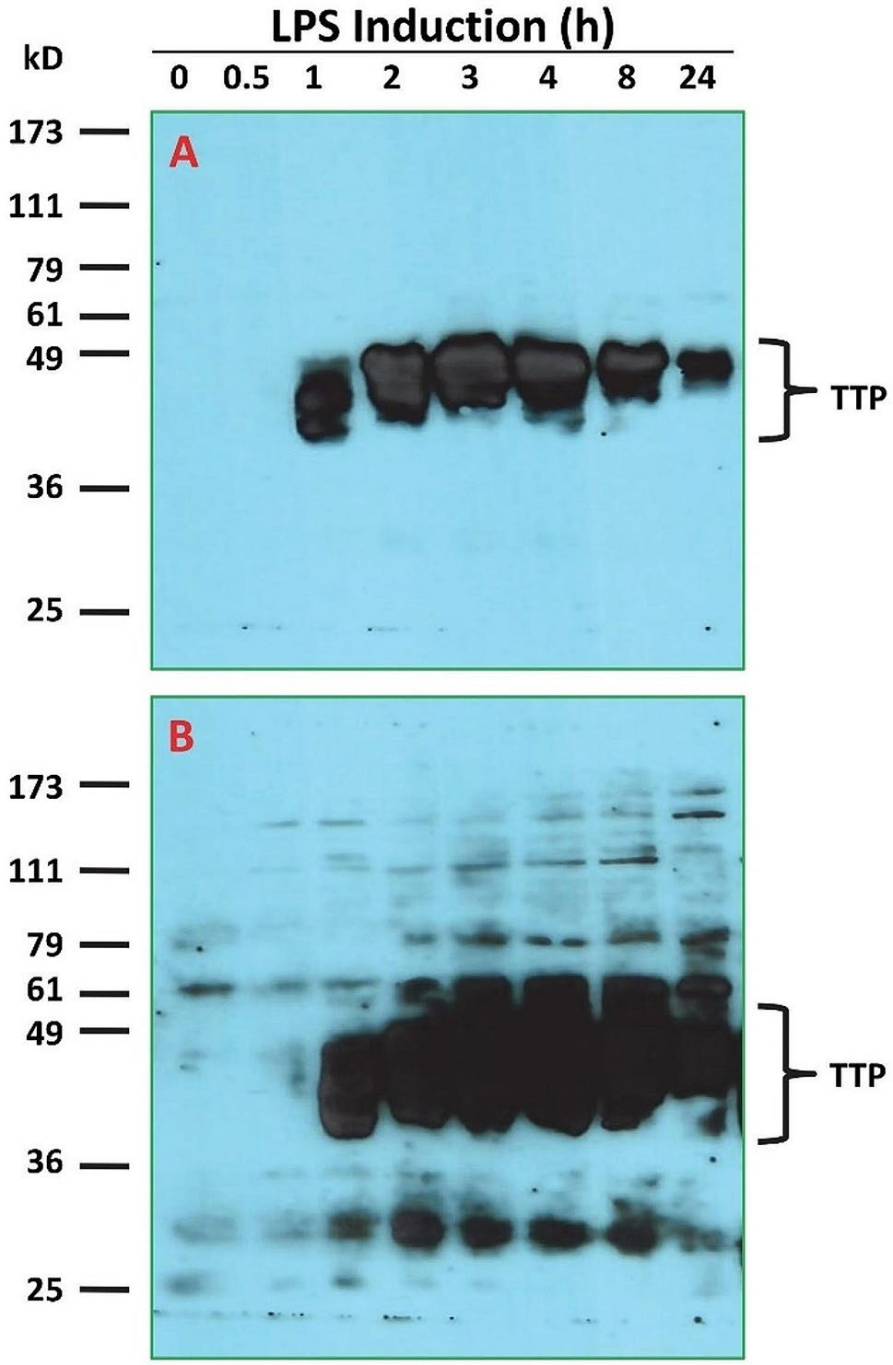
Effect of LPS on TTP protein levels in mouse macrophages. Mouse macrophages were treated with 100 ng/mL of LPS for 0, 0.5, 1, 2, 3, 4, 8, and 24 h. Proteins from a 10,000*g* supernatant (50 µg of protein per lane) were separated by10% SDS-PAGE and transferred onto nitrocellulose membranes in transfer buffer containing 0.1% SDS. The membrane was blocked with 5% nonfat dry milk in TTBS buffer and incubated with the anti-MBP-mTTP antibody (1:10,000 in blocking buffer for 1.5 h). After washed with TTBS buffer, the membrane was incubated with the affinity-purified goat anti-rabbit IgG (H + L) horseradish peroxidase conjugate (Bio-Rad, 1:10,000 in TTBS buffer for 1 h). Following washing with TTBS buffer, the membrane was incubated with Supersignal Western Blotting Detection Reagent (GE Life sciences) for 5 min before exposure to X-ray film for 30 s (Panel A) or 5 min (Panel B)

Mouse macrophages were treated with 100 ng/mL of LPS for various times. Immunoblotting was used to detect protein levels in mouse macrophages (Fig. 2). TTP antibody-reactive bands were dramatically increased by 100 ng/mL of LPS treatment for 1 h and peaked at 3 h but the protein stimulatory effect of LPS on TTP was declined after 24 h treatment (Fig. 2).

### Effect of LPS on proinflammatory gene expression

**Fig. 3:**
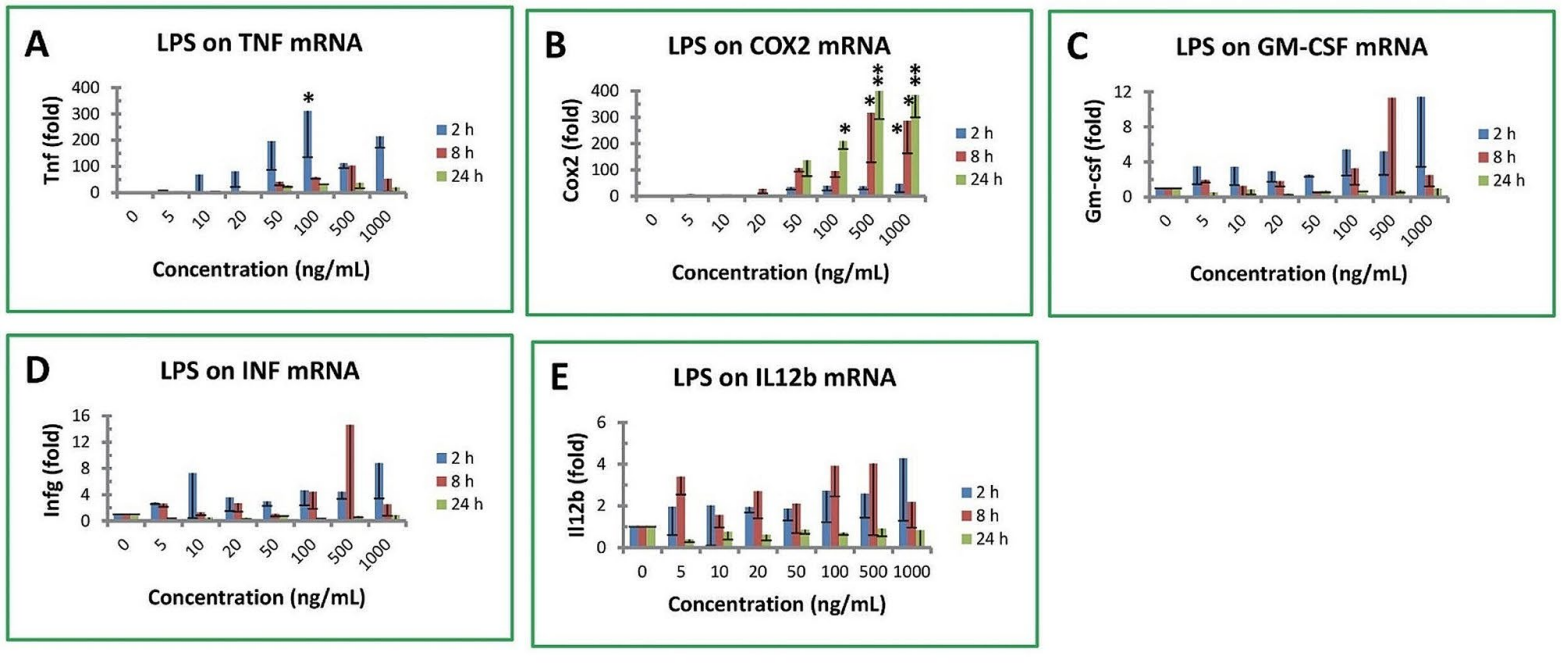
Effect of LPS on proinflammatory cytokine gene expression. (**A**) TNF mRNA, (**B**) COX2 mRNA, (**C**) GM-CSF mRNA, (**D**) IFNγ mRNA, (**E**) IL12b mRNA. RAW264.7 macrophages were treated with LPS for 2–24 h. The data represent the mean and standard deviation of three independent samples. “*” and “**” displayed in the Fig. represent significant difference between the control and the treatment at *p* < 0.05 and *p* < 0.01, respectively. The “C_T_” values of each gene in untreated cells were 17.82 ± 0.81 (Rpl32 control), 29.19 ± 2.68 (Tnf), 30.15 ± 2.38 (Cox2), 27.58 ± 2.06 (Gm-csf), 27.70 ± 2.42 (Infr) and 28.03 ± 1.89 (Il12b)

TTP is a mRNA destabilizing factor for a number of proinflammatory cytokines, such as TNF and GM-CSF [1]. Therefore, proinflammatory gene expression was investigated in mouse macrophages after treated with LPS. LPS rapidly increased mRNA levels of TNF by 12, 71, 82, 196, 311, 113 and 215 fold after 5, 10, 20, 50, 100, 500 and 1000 ng/mL treatment for 2 h, respectively (Fig. 3A). The effect of LPS on TNF gene expression markedly declined after longer treatment. The mRNA levels of TNF were only 2, 3, 6, 42, 60, 104 and 54 fold after 5, 10, 20, 50, 100, 500 and 1000 ng/mL treatment for 8 h, respectively (Fig. 3A). The mRNA levels of TNF were further reduced to 28, 35, 38 and 21 fold after 50, 100, 500 and 1000 ng/mL treatment for 24 h, respectively (Fig. 3A). It is worth pointing out that TNF mRNA levels in mouse macrophages were only statistically increased by 100 ng/mL LPS treatment for 2 h due to the large variations of the qPCR data (Fig. 3A).

LPS also strongly induced COX2 gene expression in mouse macrophages. LPS increased COX2 mRNA levels by 35, 41, 38 and 21 fold after 50, 100, 500 and 1000 ng/mL treatment for 2 h, respectively (Fig. 3B). In contrast to TNF gene expression, LPS stimulated more COX2 gene expression after longer treatment. LPS increased COX2 mRNA levels by 107, 96, 317 and 287 fold after 50, 100, 500 and 1000 ng/mL treatment for 8 h, respectively (Fig. 3B). COX mRNA levels were further increased by 137, 211, 418 and 384 fold by 50, 100, 500 and 1000 ng/mL treatment for 24 h, respectively (Fig. 3B).

The effect of LPS on the other three cytokine mRNA levels was much less than those of TNF and COX2. GM-CSF mRNA levels were increased but not statistically significant to 11 fold maximally by LPS treatment at 1000 ng/mL for 8 h or 500 ng/mL for 2 h (Fig. 3C). Similarly, INFγ mRNA levels were increased but not statistically significant to 9 fold by 1000 ng/mL for 2 h or 15 fold by 500 ng/mL for 8 h and reduced to control level in 24 h treatment (Fig. 3D). IL12b gene expression was only slightly increased but not statistically significant up to 4 fold after 500 ng/mL treatment for 8 h or 1000 ng/mL treatment for 2 h (Fig. 3E).

## Discussion

TTP family proteins play important roles in inflammation-related human diseases [12, 14]. Chemicals and agents that upregulate TTP gene expression may be able to prevent and/or treat inflammation-related diseases. It is well-known that TTP gene expression is induced by many factors like growth factors [15–17], cytokines (TNF*α*, GM-CSF, and IFN*γ*) [9, 13, 15, 17], zinc [18], plant nutritional products (cinnamon and green tea) [19, 29] and cottonseed-derived ethanol extracts and gossypol [21, 22].

It is well documented that LPS rapidly stimulates TTP gene expression at both mRNA and protein levels but less is known about its effects on other TTP family gene expression. We showed previously that TTP mRNA levels in RAW macrophages were increased by 9, 23, 11 and 39 fold after 0.5, 1, 2, and 4 h treatments with 100 ng/mL of LPS, respectively [29]. In this study, we expanded LPS treatments with a wide range of concentrations (5-1000 ng/mL) for much longer treatment time (2–24 h). We confirmed LPS-stimulation of TTP mRNA levels in the macrophages after 2 h treatment and provided new information that the magnitude of LPS-stimulation of TTP mRNA was reduced from 40 fold after 2 h treatment to 10 fold after 8 h treatment and 3 fold after 24 h treatment. These qPCR results agree with previous findings using immunoblotting and confocal microscopy methods that TTP protein levels were peaked at 1–3 h and declined after 5 h by 100 ng/mL of LPS treatment [28]. We also showed that LPS exhibited minimal effects on the mRNA levels of TTP homologues (ZFP36L1, ZFP36L2 and ZFP36L3) in the mouse macrophages.

Another important finding was that LPS markedly increased the expression of TTP-targeted proinflammatory mRNAs in mouse RAW264.7 macrophages including TNF [9] and COX2/PGES2 [32] up to 311 and 418 fold, respectively. LPS appeared to elevated the mRNA levels of GM-CSF [10], INFγ [31] and IL12 [33] up to 11, 9 and 4 fold, respectively but the differences were not statistically significant in this study. These results indicated that LPS strongly induced some TTP-mediated proinflammatory gene expression in mouse macrophages.

The results presented in this note show that LPS increased both TTP and TTP-mediated TNF and COX gene expression in the same mouse macrophages. This phenomenon agrees with the well-known fact that most of the other chemicals and agents increase both the antiinflammatory TTP gene expression and some TTP-mediated proinflammatory gene expression in cells and tissues [9, 22, 29, 37]. The regulation of some proinflammatory gene expression by the antiinflammatory TTP family proteins is proposed through a feedback inhibition [9].

## Limitations

This study focused on LPS regulation of gene expression primarily at the mRNA levels in mouse macrophages. Immunoblotting experiments were not performed on these gene products except TTP in the macrophage due to lack of good antibodies and limited resources. The fact that some of the LPS induction of gene expression related to GM-CSF, INF, IL12b and other treatments was not statistically significant could be further verified by performing more repetitions of qPCR assays to minimize data variations during statistical analyses. In addition, more needs to be done to draw a solid relationship between TTP protein and its targeted proinflammatory mRNA molecules. The disconnection between the elevated TTP mRNA and its supposed role in reducing proinflammatory mRNA levels could be addressed with subcellular compartmentation studies of TTP protein and TTP-targeted proinflammatory mRNA molecules and the structure-function relationship studies of TTP protein by post-translational modifications and regulations.

## Data Availability

The datasets generated during the current study are available in the NIH Gene Expression Omnibus (GEO) Database, accession number GSE200980 (https://www.ncbi.nlm.nih.gov/geo/query/acc.cgi?acc=GSE204820. Materials are available from the author upon request.

## List of abbreviations

COX2/PGES2: Cyclooxygenase 2/prostaglandin-endoperoxide synthase 2
GM-CSF: Granulocyte-macrophage colony stimulating factor
IL6: Interleukin 6
IL12: Interleukin 12
INFγ: Interferon gamma
LPS: Lipopolysaccharides
RPL32: Ribosomal protein L32
TNF: Tumor necrosis factor
TTP/ZFP36: Tristetraprolin/zinc finger protein 36
ZFP36L: Zinc finger protein 36-like
